# Establishment Success of the Beetle Tapeworm *Hymenolepis diminuta* Depends on Dose and Host Body Condition

**DOI:** 10.3390/insects9010014

**Published:** 2018-02-03

**Authors:** Suraj Dhakal, Sebastian Micki Buss, Elizabeth Jane Cassidy, Nicolai Vitt Meyling, Brian Lund Fredensborg

**Affiliations:** Section for Organismal Biology, Department of Plant and Environmental Sciences, University of Copenhagen, 1871 Frederiksberg C, Denmark; sura@plen.ku.dk (S.D.); qmv909@alumni.ku.dk (S.M.B.); e.cassidy@plen.ku.dk (E.J.C.); nvm@plen.ku.dk (N.V.M.); blf@plen.ku.dk (B.L.F.)

**Keywords:** *Hymenolepis diminuta*, *Tenebrio molitor*, invertebrate–parasite model, cysticercoid establishment, host immune competence

## Abstract

Parasite effects on host fitness and immunology are often intensity-dependent. Unfortunately, only few experimental studies on insect-parasite interactions attempt to control the level of infection, which may contribute substantial variation to the fitness or immunological parameters of interest. The tapeworm *Hymenolepis diminuta*—flour beetle *Tenebrio molitor* model—has been used extensively for ecological and evolutionary host–parasite studies. Successful establishment of *H. diminuta* cysticercoids in *T. molitor* relies on ingestion of viable eggs and penetration of the gut wall by the onchosphere. Like in other insect models, there is a lack of standardization of the infection load of cysticercoids in beetles. The aims of this study were to: (1) quantify the relationship between exposure dose and establishment success across several *H. diminuta* egg concentrations; and (2) test parasite establishment in beetles while experimentally manipulating host body condition and potential immune response to infection. Different egg concentrations of *H. diminuta* isolated from infected rat feces were fed to individual beetles 7–10 days after eclosion and beetles were exposed to starvation, wounding, or insertion of a nylon filament one hour prior to infection. We found that the establishment of cysticercoids in relation to exposure dose could be accurately predicted using a power function where establishment success was low at three lowest doses and higher at the two highest doses tested. Long-term starvation had a negative effect on cysticercoid establishment success, while insertion of a nylon filament and wounding the beetles did not have any effect compared to control treatment. Thus, our results show that parasite load may be predicted from the exposure dose within the observed range, and that the relationship between dose and parasite establishment success is able to withstand some changes in host body condition.

## 1. Introduction

Insects and other arthropods are important model organisms to investigate host–parasite interactions [1,2,3,4], and they may in the future complement and partly replace vertebrate models, which experience several ethical, legislational, and logistical constraints. An important pre-requisite for the use of insects in parasite research is an accurate prediction of parasite load in experimentally infected hosts in relation to the dose of infective stages. Knowledge of parasite load is needed to determine intensity-dependent effects on both host and parasite fitness [5,6,7]. The relationship between infection dose and the number of established parasites can be used to indicate the effect of host body condition and immune competence on parasite establishment success at different levels of parasite exposure. Thus, insect-parasite models hold the potential to test general concepts related to interactive effects between nutrition, innate immune function, and infection biology.

The rat tapeworm *Hymenolepis diminuta* (Cestoda) and its beetle host *Tenebrio molitor* (Coleoptera) is a well-known host–parasite model to study ecological interactions between helminths and their hosts [8,9]. Furthermore, this insect–parasite model has been used for screening of potential pharmacological compounds as an alternative for vertebrate testing [4,10], and to investigate host-pathogen interactions in an ecotoxicological context [11]. Transmission success of *H. diminuta* from the rodent definitive host to *T. molitor* depends upon the ingestion of viable eggs present in rat feces, penetration of the gut wall by the oncosphere larva, and establishment of cysticercoids in the hemocoel of the beetle [12]. So far, no standardized infection protocol to control the number of *H. diminuta* in individual *T. molitor* has been established, and most experiments utilizing the *T. molitor*/*Tribolium confusum*–*H. diminuta* model display a large variation of cysticercoid load among individual beetles within and across experiments [8,13,14,15]. In addition, there is a lack of studies examining the proportion of cysticercoid establishment in *T. molitor* in relation to the exposure dose of *H. diminuta* eggs over a wide range of doses.

Besides exposure dose, manipulations of host body condition that affect immune function may have effects on establishment success of *H. diminuta* cysticercoids. RNAi knockdown of insect antimicrobial peptides (AMPs) showed an increase in midgut parasite infections [16] while nutritional status has been found to affect immune response, with nutritional deprivation resulting in a down-regulation of immune effector systems [17]. Hosts have also demonstrated a modulation of nutritional intake in response to parasite infection [18]. In the beetle host *T. confusum*, *H. diminuta* establishment reached a plateau with increasing exposure time. In contrast, beetle gender did not make any difference in susceptibility towards infection but increasing beetle age markedly decreased the parasite load [8].

In previous efforts to develop a standardized infection protocol for the *H. diminuta*–*T. molitor* system, we found that the establishment success of cysticercoids was not directly proportional to the exposure dose [19]. Hence, there was a relatively low proportion of established *H. diminuta* cysticercoids in *T. molitor* with low egg exposure (<100 eggs per beetle) compared to higher egg doses. Based on those observations we hypothesize that the beetle may resist successful parasite establishment at low exposure dose by inducing an effective immune response to infection. To test this hypothesis we manipulated beetle host condition in ways that would either stimulate or reduce an immune response to infection, and compared the number of cysticercoids that developed in the hemocoel of hosts in relation to exposure dose.

The objectives of this study were therefore twofold. The first aim was to investigate the dose-dependent establishment success of cysticercoids over a range of different exposure doses of *H. diminuta* eggs using the intermediate host *T. molitor* to enable predictive experimental infections with resulting infection load within the range of infections observed in natural host populations [20]. The second aim was to test the effect of manipulation of host body condition on establishment success of *H. diminuta* by nutritional deprivation, wounding and insertion of a nylon filament. Starved hosts were expected to be immunocompromised [17], which may increase establishment success, whereas activating the immune response by insertion of a nylon filament [21,22] may decrease establishment success of *H. diminuta* cysticercoids due to the mounting of the immune system. 

## 2. Materials and Methods

### 2.1. Management and Infection of Tenebrio Molitor

*Tenebrio molitor* cultures were reared on oatmeal and egg white protein (50:1)supplemented by fresh potato (weekly) and maintained in plastic boxes (12 × 18 cm) incubated in the dark in a climate cabinet at 26 °C [10]. At pupation, individuals were collected and placed in separate boxes for adult emergence. Adults emerging within a 4-day window were collected and maintained as above at low density (approximately 50 beetles per box) for another 7 days before infection. Adult *T. molitor* were infected with *H. diminuta* eggs collected from feces of infected rats (*Rattus norvigecus*—Wistar strain) stabled at the University of Copenhagen, Denmark (Animal permission No. 2010/561-1914, Section C10). The infected rat feces were collected every week and stored at room temperature until use (2 days maximum), and the eggs were therefore 2–10 days old at the time of use. Ten grams of rat feces were soaked in 25 mL tap water for one hour, stirred with a wooden spatula to make a uniform paste, sieved through a double layer of cotton gauze (1 × 1 mm) followed by sieving through 100, then 62-micron metal sieves. The eggs collected on the 62-micron sieve were suspended in tap water in 50 mL centrifuge tubes, and centrifuged (Universal 16R, Gemini^BV^, Apeldoorn, The Netherlands) at 300 g for 3 min. The supernatant was discarded and the sediment was used for egg dose preparation [10] by placing 10 µL of egg suspension on a glass slide and counted under a compound microscope (100×). Egg viability was tested on an additional random sample of the egg suspension, by placing a droplet of the suspension on a glass slide, and a cover glass was gently pressed down on the eggs until the outer egg capsule ruptured mimicking the mechanical rupture of eggs by beetle mandibles during ingestion. This procedure activated >95% of the oncosphere larvae in the sample. 

### 2.2. Establishment Success with Different Egg Concentrations

Seven to ten day old beetles were starved for 72 h prior to experimental exposure to *H. diminuta* eggs. Thirty beetles were exposed individually to 10 µL of egg suspension presented on glass slide of one of five different concentrations (mean eggs per 10 µL ± SE; 34 ± 1, 69 ± 2, 129 ± 5, 253 ± 9, and 443 ± 7) for 1 h in a dark condition. Beetles consuming all 10 µL of egg suspension were transferred to a box (12 × 18 cm) and placed in a dark incubator at 26 °C, while beetles that did not consume the entire suspension volume were removed from the experiment. Following the exposure, the glass slides were examined under microscope (100×) and the numbers of eggs remaining on each slide were counted to estimate the approximate number of eggs that were ingested by each beetle. At 15 days post infection, 15 beetles from each exposure group were dissected individually in phosphate buffered saline (PBS) and cysticercoids were counted under dissection microscope (40×) to measure the establishment success. This experiment was repeated on three separate occasions.

A separate experiment using four different concentrations (mean eggs per 10 µL ± SE; 40 ± 2,81 ± 3, 123 ± 2, and 163 ± 3) of *H. diminuta* eggs were used to infect *T. molitor* as described above to evaluate if predictions for parasite load can be made. As the first experiment showed relatively low establishment success of cysticercoids at the low doses, the second experiment focused on four doses within the lower range of egg concentrations. This experiment was repeated four times with 20 beetles in each exposure group.

### 2.3. Host Body Manipulation

Based on the above experiments, we tested the potential role of the host body manipulation on the establishment success of *H. diminuta* at two different doses. Thus, *T. molitor* beetles were treated in four different ways one hour prior to infection with *H. diminuta* eggs to alter the host body condition; (1) starvation, (2) insertion of a nylon filament, (3) wounding of the sternum with a sterilized needle, and (4) an untreated control group. These different treatments were chosen based on their potential to alter the immune system of *T. molitor* in different ways that would either facilitate [22], or reduce an immune response [17]. In the starvation treatment, beetles were kept with access only to tap water seven days prior to and four days after exposure to the *H. diminuta* egg suspensions. In treatment (2) beetles were chilled for 3–5 min on ice, a wound was made by penetrating the cuticle with metal a pin (around 0.2 mm diameter, swabbed with 70% ethanol) between the third and fourth sternite, after which a 2 mm fragment of nylon monofilament (0.127 mm diameter, Rio, Powerflex tippet, Idaho Falls, Idaho USA) swapped with 70% ethanol was inserted inside the body cavity [23], one hour prior to exposure. The wounded group was treated the same way as treatment (2), but no nylon filament was inserted into the wound in order to evaluate effect of the injury itself. The control group did not receive any treatment.

All beetles from the treatment group (1) were starved for seven days and treatments (2), (3), and (4) were starved for three days prior to *H. diminuta* infection to ensure maximum consumption of eggs. All the treatments were performed with two different infection doses; “Low” (approximately 80 eggs) and “High” (approximately 200 eggs) based on the establishment success in the experiments above. After exposure, all glass slides were examined for remaining eggs and beetles were discarded if more than 15 and 25 eggs were left on the slide from low and high dose, respectively. Beetles were subsequently kept in the incubator (26 °C) and dissected after 15 days post infection to quantify cysticercoids as described above. The experiment was repeated three times. 

### 2.4. Phenoloxidase (PO) Activity

To assess the effect on immunocompetence at the time of exposure, 20 beetles per group were maintained in the same four different treatment groups as above, but not exposed to eggs.

Hemolymph was collected from beetles at the same time as exposure occurred in the infection treatments, that is, 7 days after the starvation treatment commenced, and an hour after the wounding and nylon filament treatments took place. Nylon filament, wounded and control beetles were starved (with access to water) for 3 days before hemolymph extraction. Beetles were kept on ice to incapacitate them before extraction. A wound was made between the pronotum and thorax on the ventral surface of the beetle, and 2 μL of hemolymph was collected with a micropipette, and added to 18 μL of cooled PBS. Samples were kept on dry ice before being stored at −80 °C.

Phenoloxidase (PO) activity was measured spectrophotometrically as the rate of conversion of L-DOPA to dopachrome by PO. The hemolymph/PBS mixture from two individuals within a treatment was pooled for PO measurement. 30 μL of hemolymph/PBS mixture was added to a well containing 30 μL of PBS, to which 100 μL of L-DOPA was added and mixed thoroughly. The reaction was allowed to proceed in SpectraMax® i3 (Molecular Devices, San Jose, CA, USA) for 60 min with readings at 490 nm taken every 2 min at 28 °C. Enzyme activity was measured as the *V*_max_ (the slope of the reaction during the linear phase).

Protein concentration was also determined for the hemolymph/PBS mixture using the Bradford protein concentration assay. 250 μL of Bradford reagent was added to 5 μL of hemolymph/PBS and mixed thoroughly in a 96 well plate. Absorbance was measured at 595 nm and protein content values obtained using bovine serum albumen (Sigma-Aldrich^®^, St. Louis, MO, USA) as a standard. PO activity is expressed as *V_max_* (slope of the reaction) per μg of hemolymph protein.

### 2.5. Statistical Analyses

Statistical analyses were performed using SAS^®^ version 9.4 (SAS institute Inc., Cary, NC, USA). The level of significance was set at α = 0.05. The number of cysticercoids established with different exposure doses, between different treatment groups, and number of uneaten eggs were modeled using generalized linear mixed models (PROC GLIMMIX procedure in SAS), fitting negative binomial distributions. Repetition of the experiments was included as a random effect in the models. Prevalence of cysticercoids infection and proportion of uneaten eggs were calculated by using a chi-square test.

## 3. Results

### 3.1. Establishment Success with Different Egg Concentrations

**Figure 1:**
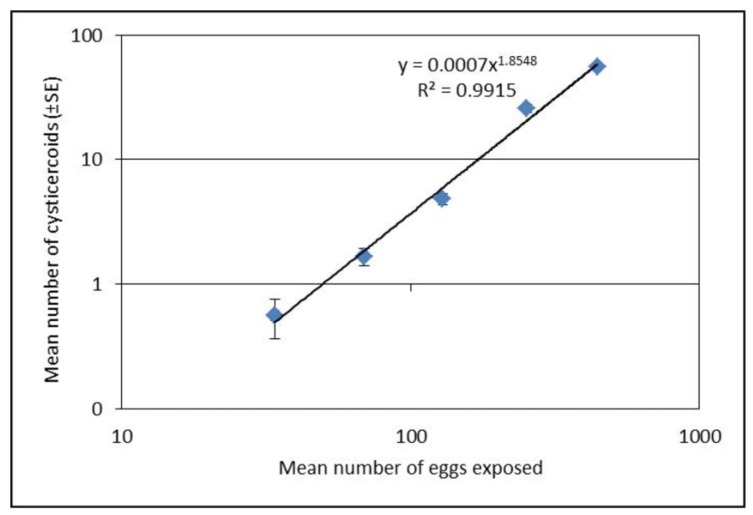
Mean number of cysticercoids (±SE) after exposure with five different concentrations of eggs based on dissection of 45 *Tenebrio molitor* per dose (three repetitions each with 15 beetles). The relationship between egg dose and establishment of cysticercoids was best explained by a power function: (Number of established cysticercoids = 0.0007 × egg dose ^1.8548^, *R^2^* = 0.9915). Presented values in the figure are on logarithmic scale.

**Figure 2:**
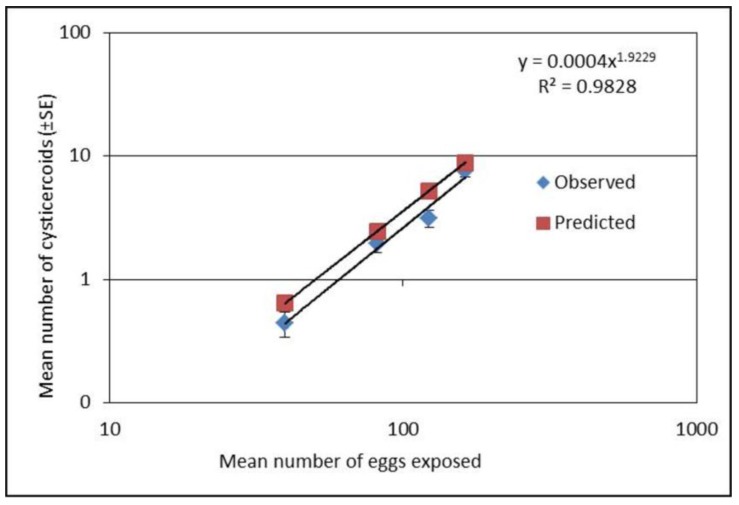
Mean number of cysticercoids (±SE) after exposure with four different concentrations of eggs. The numbers of *Tenebrio molitor* used for cysticercoids count were 68, 56, 59 and 60 for the four doses, respectively. Predicted values were derived using formula (*y* = 0.0007 *x*^1.8548^; Figure 1). Presented values in the figure are on logarithmic scale.

Establishment of *H. diminuta* cysticercoids in *T. molitor* was significantly different among exposure groups for both experiments (*F*_4,218_ = 182.95, *p* < 0.0001; Figure 1 and *F*_3,237_ = 47.85, *p* < 0.0001; Figure 2) with the highest dose displaying the greatest number of cysticercoids. Establishment success as function of exposure dose could be described as a power function (Figure 1). Although the established cysticercoid numbers in the second exposure experiment (Figure 2) were slightly lower than the predicted values, the slopes were similar. Our results showed that a lower proportion of eggs were left on slides (22, 20, 22, 9 and 8% for the five doses, respectively) (*x^2^* = 26.61, *df* = 4, *p* = <0.0001) and a higher proportion of established cysticercoids (2, 2, 4, 10 and 13%) with an increased exposure dose. Also, the prevalence of infection was relatively lower (between 1/3 and 2/3 infection success) when beetles were exposed to less than 100 eggs (Table 1).

### 3.2. Host Body Manipulation

**Figure 3:**
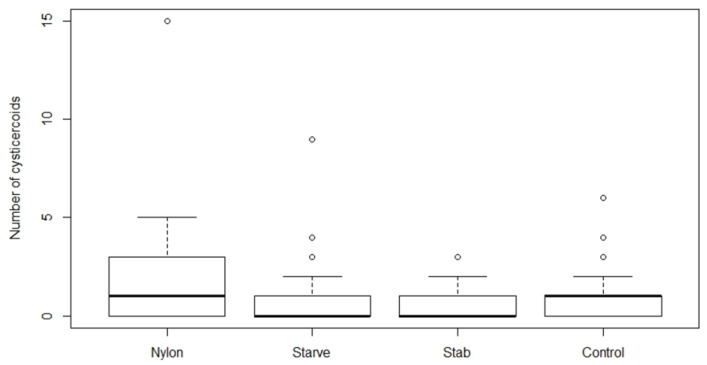
Number of cysticercoids developed after exposing *Hymenolepis diminuta* eggs (79 ± 6 SD) with different treatment strategies on *Tenebrio molitor* host. The average number of *T. molitor* hosts used for counting cysticercoid was 27, 43, 25 and 30 for nylon, starvation, wounding, and control group, respectively. The nylon group refers to the insertion of a 2 mm nylon filament into the body cavity. Starvation refers to deprivation of food for 10 days, while the wounding group served to test the effect of the injury on the abdomen without the insertion of the nylon filament. The control group received ad libitum food and did not receive any treatment.

**Figure 4:**
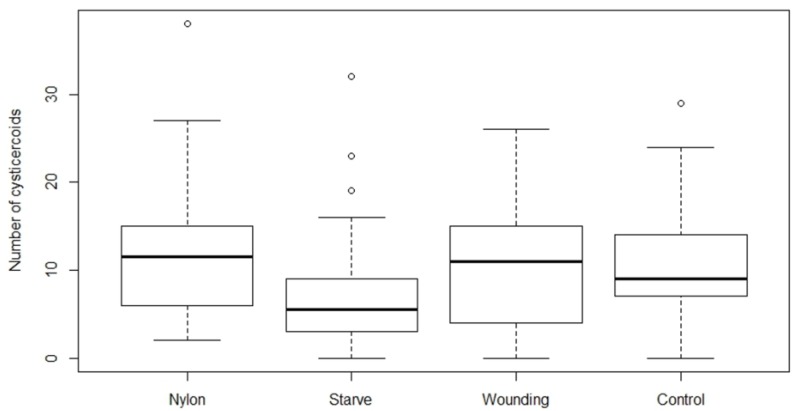
Number of cysticercoids developed after exposing *Hymenolepis diminuta* eggs (199 ± 11 SD) with different treatment strategies on *Tenebrio molitor* host. The average number of *T. molitor* hosts used for counting cysticercoid was 30, 42, 21 and 33 for nylon, starvation, wounding, and control group respectively. The nylon group refers to the insertion of a 2 mm nylon filament into the body cavity. Starvation refers to deprivation of food for 10 days, while the wounding group served to test the effect of the injury on the abdomen without the insertion of the nylon filament. The control group received ad libitum food and did not receive any treatment.

There was a significant effect of treatment on cysticercoid establishment at the low dose (80 eggs) of *H. diminuta* eggs (*F*_3,124_ = 2.71, *p* = 0.0481; Figure 3), and a marginally insignificant effect of treatment at the high dose (200 eggs) (*F*_3,120_ = 2.61, *p* = 0.0546; Figure 4). Starved beetles receiving high egg exposure harbored a significantly lower number of cysticercoids compared to the control group(*p* = 0.0233), and also compared to the nylon filament group (*p* = 0.0173). There was a significant difference between the number of uneaten eggs left on glass slides in both the high (*F*_3,98_ = 15.04, *p* = <0.0001) and low (*F*_3,76_ = 5.17, *p* = 0.0026) dose treatments. Among the four treatment groups, the starved beetles left significantly fewer eggs uneaten on the slides compared to the other treatment groups. However, the starved beetles also displayed the lowest establishment of cysticercoids (Table 2). Thus, the proportion of consumed *H. diminuta* eggs establishing as cysticercoids in starved beetles was reduced by 14 and 37% at high and low exposure, respectively, compared to the control group. The establishment of cysticercoids in the starvation group was 57 and 40% reduced compared to beetles with an inserted nylon filament. Phenoloxidase activity (*V_max_*, slope of reaction ±SE) per μg of protein content in the hemolymph of tested individuals from nylon inserted, starvation, wounding and control group were 0.5 ± 0.07, 0.48 ± 0.04, 0.53 ± 0.08 and 0.64 ± 0.04, respectively and was not affected by the treatments (*F*_3,38_ = 1.207, *p* = 0.322).

## 4. Discussion

We found that establishment success of *H. diminuta* cysticercoids in *T. molitor* hosts is density dependent, and only the starvation had an effect among the body manipulation treatments. The relationship between exposure dose and parasite establishment could best be explained by a power equation, i.e. the relationship was non-linear. Thus, it was possible to predict the rate of cysticercoids establishment based on the exposure dose the beetles received although the exact number of cystercoids can vary among beetle batches. The exposure protocol presented here is therefore a useful method for predicting infection load in this host–parasite model system within a range encountered in a natural population [20], which can be valuable in future studies.

Interestingly, the proportion of established cysticercoids was higher at concentrations above 100 eggs per 10 µL compared to lower concentrations. Lower proportional establishment of cysticercoids at the low concentrations of <100 eggs per 10 µL, may suggest that *T. molitor* are more successful in resisting infection at relatively low doses. At the low dose, overall infectivity was low (29%), and most infected hosts developed very few cysticercoids (usually 1 or 2). Alternatively, the lower establishment success at lower doses could have a methodological explanation as a higher proportion of uneaten eggs was found for the lower exposure concentration (<100 eggs). Thus, there is a decreased proportion of fecal debris with the lower concentration of eggs, which may have altered the viscosity of the egg suspension and negatively affected ingestion of eggs. Therefore, counting of uneaten eggs gives a more accurate estimation of dose expressed as eggs that were actually consumed. In addition to the above, at low egg doses there may be a greater probability that the eggs fail to cause an infection by random chance alone. This would result in a positive relationship between the mean number of cysticercoids per beetle and the prevalence of infection, which is a common pattern observed among parasite populations under natural conditions.

The host may utilize various defense mechanisms to prevent or combat early infection. A common immune response of insects to macroparasites is via the PO cascade pathway encapsulating and melanizing the parasite [24]. While other studies suggest an immune activation from nylon implants [21,22], neither nylon nor wounding affected the cysticercoid number in our study. However, we did not observe cysticercoids of *H. diminuta* melanized within the hemocoel on any occasion in this study, and previous studies have suggested that mature cysticercoids may actively hinder encapsulation and melanization [25]. Therefore, an effect of the PO pathway on parasite establishment in this system would probably only be observed at an early stage of cysticercoid development. Insertion of a nylon filament in our study was conducted to prime the immune response to parasite infection. However, we did not observe any effect of nylon insertion on the number of cysticercoids, suggesting that establishment is not restricted by the PO pathway. There might be a tradeoff between coping with starvation and immune activation as beetles are starving for three days between nylon insertion and exposure, and our finding also revealed the same level of PO activity at the time of exposure. It is also possible that *H. diminuta* downregulates the expression of genes related to the PO pathway as indicated in *Tribolium confusum* [26].

While immune priming did not alter parasite establishment, starvation significantly decreased cysticercoid establishment. Short-term starvation and host stress factors are known to play a role in the host immune response [17], which may be expected to result in higher susceptibility to infection. However, in our experiment, starvation decreased the parasite establishment, despite that starved hosts were consuming a greater number of eggs in comparison to nylon insertion, wounding and control groups. Although modulation of host nutrient intake occurs in response to parasite infection [18,27], this was unlikely in this study due to starvation. Since the beetles are in a nutrient deprived state at the time of exposure and for several days afterwards, they might be too nutrient deficient for efficient parasite establishment and development, which might lead to poor establishment success. The early development of the cysticercoid in the body cavity of the host is characterized by rapid growth and development within the first 7–10 days of infection after which it slows down and completes its development by approximately 14 days, but this is highly dependent on the environmental temperature [28]. During the time of rapid development, the host experiences a significant loss in fecundity even when infected with approximately 80 cysticercoids, possibly reflecting a high energetic demand of the parasites during this period [29]. Starvation and consequences of starvation might affect the parasite development and resulting establishment success explaining the reduced number of cysticercoids observed in our study, but further experiments are needed to investigate that possibility further.

Host digestive enzymes also play a crucial role in *H. diminuta* egg hatching inside the digestive tract of beetles [12]. Long periods of starvation may lead to altered secretion of host digestive enzymes, alteration of gut retention time and poor egg hatching. Beside this, starvation might lead to alterations of the gut microbiota, which also may have an important role in the hatching parasite eggs. This is well known from studies on helminths of vertebrate hosts [30,31], but it has also recently been confirmed in *T. molitor* where beetles, which microbiota has been disrupted by treatment with antibiotics, display a significantly reduced number of established *H. diminuta* cysticercoids compared to an untreated control group [32].

## 5. Conclusions

Our results show that the number of established cysticercoids increased with higher exposure dose in a non-linear fashion and that the variation in the infection load at each dose was limited. Furthermore, starvation of beetles had a negative effect on cysticercoid establishment. The exposure method and dose of eggs described here therefore serves as a good and predictable proxy for establishment of cysticercoids within the observed range in the beetle host. This finding opens the opportunity to further examine the relationship between parasite load and various host fitness and immune parameters.

## Figures and Tables

**Table 1:** Mean (±SE) number of exposed and uneaten *Hymenolepis diminuta* eggs and number and prevalence of cysticercoids developed in *Tenebrio molitor* after exposure to five different concentrations. Different letters within each column represent significant differences between treatment groups (*α* = 0.05). # refers significant different within column (*x*^2^ = 89.68, *df* = 4, *p* = <0.0001).

No. of Exposed Eggs (±SE)	No. of Uneaten Eggs (±SE)	No. of Cysticercoids (±SE)	Prevalence % of Infection #
34 ± 1.3	7 ± 1.15	0.56 ± 0.2 ^a^	29
69 ± 1.87	14 ± 3.24	2 ± 0.26 ^b^	67
129 ± 5.02	28 ± 4.38	5 ± 0.49 ^c^	96
253 ± 9.4	24 ± 3.6	26 ± 2.05 ^d^	98
443 ± 7.07	36 ± 6.29	56 ± 4.02 ^e^	96

**Table 2:** Mean (±SE) number of uneaten *Hymenolepis diminuta* eggs and cysticercoids developed in *Tenebrio molitor* for nylon, starvation, wounding, and control group after exposed with two different doses of *H. diminuta* eggs (low dose and high dose). The nylon group refers to the insertion of a 2 mm nylon filament into the body cavity. Starvation refers to deprivation of food for 10 days, while the wounding group served to test the effect of the injury on the abdomen without the insertion of the nylon filament. The control group received *ad libitum* food and did not receive any treatment. Different symbols/letters (*, #, a, A) within each row represent statistical significance (α = 0.05). No difference on prevalence % of infection (*x^2^* = 3.85, *df* = 3, *p* = 0.277 for low dose and *x^2^* = 3.94, *df* = 3,*p* = 0.266 for high dose).

	Nylon	Starved	Wounding	Control
Low dose (79 ± 1.86)				
Uneaten eggs	13 ± 3.03 *	5 ± 0.97 **	11 ± 2.02 *	11 ± 2.62 *
Cysticercoids	2 ± 0.58 ^a^	0.88 ± 0.25 ^b^	0.76 ± 0.2 ^b^	1.05 ± 0.23 ^a,b^
Prevalence % of infection	59	40	44	57
High dose (199 ± 3.29)				
Uneaten eggs	19 ± 3.92 ^#^	4 ± 0.83 ^##^	16 ± 3.57 ^#^	14 ± 1.58 ^#^
Cysticercoids	11.73 ± 1.47 ^A^	7.04 ± 1.03 ^B^	10.8 ± 1.69 ^A, B^	10.51 ± 1.21 ^A^
Prevalence % of infection	100	90	86	91
